# Iatrogenic injury to long thoracic nerve following thoracotomy for right thoracic scoliosis in Marfan syndrome: a case report

**DOI:** 10.1186/s13256-021-02755-z

**Published:** 2021-03-26

**Authors:** Saeid Safaei, Ahmadreza Mirbolook, Parisa Azimi, Mirbahador Athari, Farhad Hamzehzadeh, Taravat Yazdanian

**Affiliations:** 1 Department of Spine Surgery, Milad General Hospital, Tehran, Iran; 2 Department of Orthopedic, Imam Hossain Medical Center, University of Shahid Beheshti Medical Sciences, Shahid Madani Street, Tehran, Iran; 3 Neuroscience Research Center, Shahid Beheshti University of Medical Sciences, Arabi Ave, Daneshjoo Blvd, 19839-63113 Velenjak, Tehran Iran; 4 Department of Orthopedic Surgery, Milad General Hospital, Tehran, Iran; 5 School of Medicine, Capital Medical University, Beijing, China; 6 Parisa Azimi Neuroscience Research Center, Shahid Beheshti University of Medical Sciences, Arabi Ave, Daneshjoo Blvd, 19839-63113 Velenjak, Tehran Iran

**Keywords:** Marfan syndrome, Thoracic scoliosis, Long thoracic nerve, Winged scapula, Long thoracic nerve injury

## Abstract

**Background:**

Patients with Marfan syndrome commonly require spinal deformity surgery. The purpose of this case report is to present a rare thoracotomy complication. We present the management of such a patient.

**Case summary:**

In a known case of Marfan syndrome, an 18-year-old Persian man was admitted to our hospital with scoliosis. The patient underwent radiological examinations, and thoracic scoliosis of 70° was diagnosed. A right thoracotomy for anterior spinal fusion from the sixth rib and posterior spinal fusion were performed successfully. Two months later, he was readmitted because of winging of the right scapula due to serratus anterior palsy. Electromyography and nerve conduction velocity confirmed long thoracic nerve injury. Conservative treatment was provided. Ultimately, the patient recovered completely in the last follow-up visit 6 months after the surgery.

**Discussion:**

This is the first report of ipsilateral winged scapula after thoracotomy. Attention needs to be paid to surgical techniques in patients with Marfan syndrome.

## Introduction

Marfan syndrome (MFS) is an illness of connective tissue, mainly involving the musculoskeletal, ocular, and cardiovascular systems [1], with an incidence of 2–3 per 10,000 population [1, 2]. Scoliosis occurs in approximately 50–70% of patients with MFS and differs from idiopathic adolescent scoliosis with regard to curve pattern, progression, and symptoms [3]. Overall, cases with curves greater than 40–50° require surgical intervention that may be associated with surgical complications such as scapular winging [4].

Scapular winging is a painful and progressive disease. Weakness of scapular mechanics can cause problems with elevating the arm and lifting objects [5]. It is the result of numerous causes, most often resulting in nerve injury and paralysis of either the serratus anterior from long thoracic nerve injury causing medial winging or trapezius dysfunction from spinal accessory nerve injury causing lateral winging [5]. The incidence of long thoracic nerve injury was reported to be between 0.0026% and 0.21% [5]. We report a rare case of Marfan syndrome with scapular winging complication after thoracotomy for right thoracic scoliosis. The clinical summary, imaging findings, and surgical procedures are discussed

## Case presentation

**Fig. 1:**
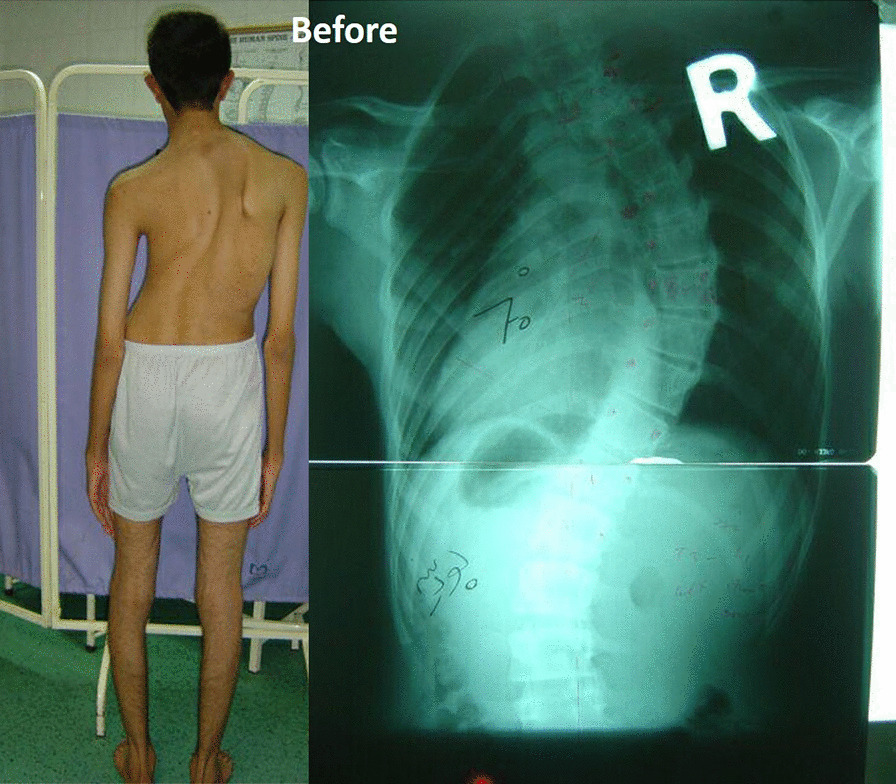
Initial radiograph. Patient with Marfan syndrome and with right thoracic scoliosis of 70°

**Fig. 2:**
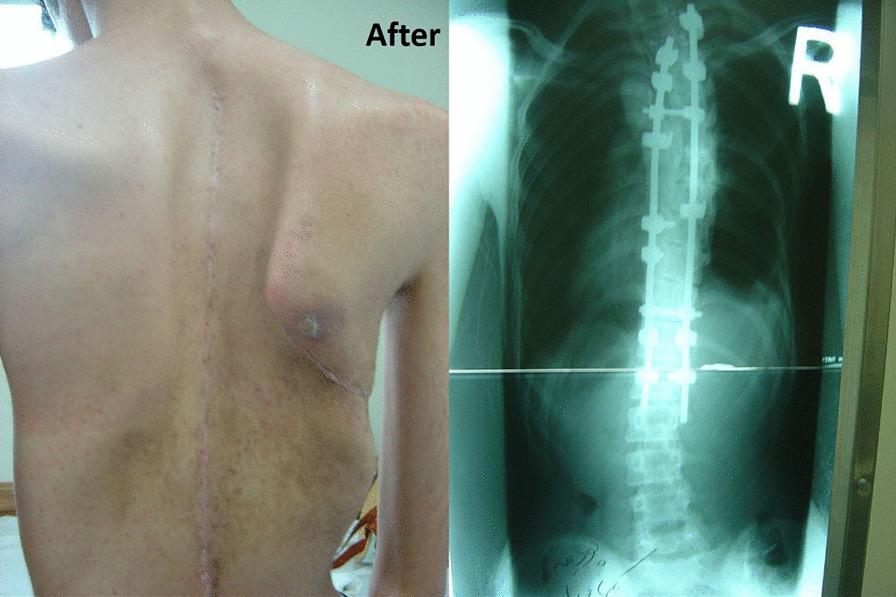
Winging of the right scapula due to serratus anterior palsy, in pushing posture in the 2 months after second surgery

**Fig. 3:**
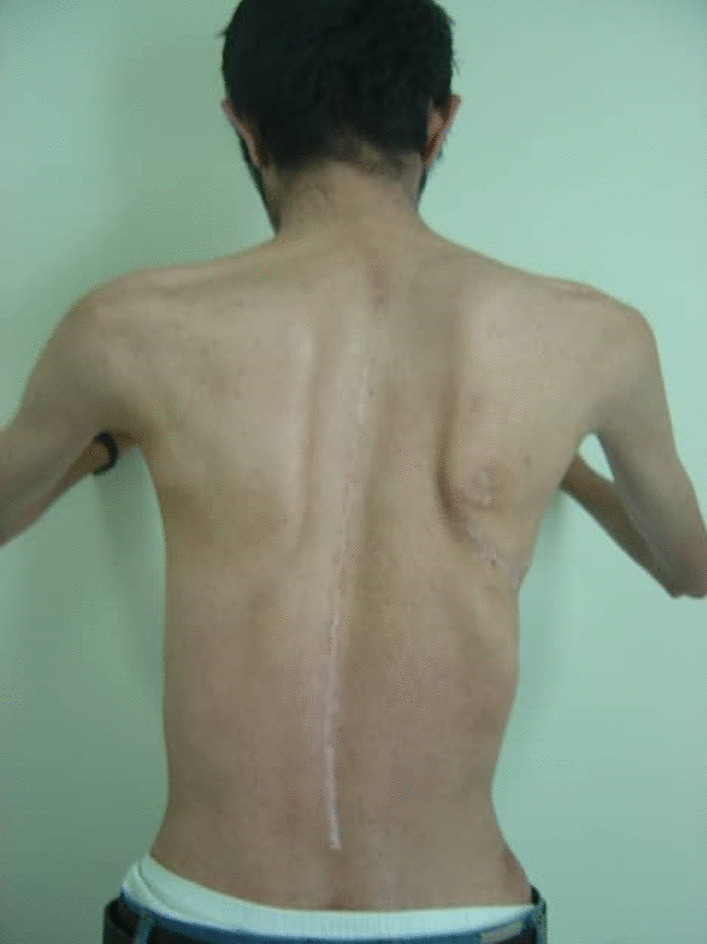
Latest photograph. Winging of the right scapula in the last follow-up visit 6 months after surgery

An 18-year-old Persian man (60 kg, 180 cm) with a case of Marfan syndrome (MFS) identified from family history and genetic assessment was referred to our center. He presented mainly with right thoracic scoliosis of 70° and excessive joint laxity, without any previous disease history (Fig. 1). Scoliosis of 70° and the deteriorating condition of our patient led to the decision to perform surgery. In April 2016, a right thoracotomy for anterior spinal fusion (ASF) was performed from the sixth rib under general anesthesia in the left lateral decubitus position. After 3 days, posterior spinal fusion and instrumentation (PSF) was done successfully. He was discharged with a total contact body brace. On the 2 months postoperative visit, he complained of weakness of the right upper limb during overhead activities. On physical examination, the right upper limb was intact for both sensory and motor innervation, but the right scapula was winged and he was not able to flex his arm forward over 60° (Fig. 2). Electromyography (EMG) and nerve conduction velocity (NCV) confirmed long thoracic nerve injury. A conservative treatment, physiotherapy, was provided for 3 months. Ultimately, the patient recovered completely in the last follow-up visit 6 months after the surgery. Electromyography and nerve conduction velocity revealed a return to normal position. However, scapula is more prominent than normal due to the hyperlaxity of MFS (Fig. 3).

## Discussion

This is the first case in the literature with the unusual complication of scapular winging following thoracotomy in MFS. Scoliosis is the most common major skeletal deformity encountered in MFS patients that requires intervention. Bracing can be considered in patients with mild scoliosis. However, most patients with MFS will have significant curve development and progression that ultimately warrants surgical intervention. Overall, cases with curves greater than 40–50° or with associated abnormal sagittal curvature deformities require surgical intervention. Scoliosis in patients with MFS can be corrected similarly to the way it is corrected in idiopathic scoliosis. These patients often require anterior fusion and posterior instrumentation and fusion, which was confirmed in our patient [4].

The serratus anterior is a broad muscle that originates in the first to ninth ribs. The serratus anterior muscle function is to stabilize the scapula against the chest wall while elevating the arm [6]. The serratus anterior and trapezius muscles rotate the scapula concurrently so that the arm can be raised to the vertical position [6].

Several reasons for long thoracic nerve damage have been reported in literature, such as closed trauma, stretching, compression, traction, penetrating injury, direct extrinsic force, inappropriate surgical technique, electrocution, chiropractic manipulation, and various sports-related injuries. Also, scapular winging can result from repetitive or sudden external biomechanical forces [4, 5, 7, 8]. In the anterior approach, when a patient is under general anesthesia, there is less control of the shoulder girdle muscles. Also, passive adduction of the arm may cause the scapula to shift anteriorly, for example, when a patient’s arm is raised and crossed over his or her chest. This could cause creasing of the muscle and compression of the long thoracic nerve [9]. Especially in MFS, because of joint laxity, intraoperative stretching on the ribs and scapula can cause injury to the nerve that is greater than in other patients.

In this study, it seems that, due to thoracotomy from the sixth rib and scapular traction during surgery, iatrogenic injury to the long thoracic nerve and winged scapula was observed. The recommended treatment for winged scapula following surgery is initially nonsurgical, that is, physical therapy. Different results have been attained using braces, standard arm slings, and other orthotics. Some propose that nonoperative treatment may be the best course of action for patients with minimal symptoms [8], which is in line with this study. This case was unique insofar as there is no prior report of this in a Marfan patient undergoing scoliosis repair. Hence, it may play a role in medical research and evidence-based medicine. Importantly, it informs the decision-making process, allowing other clinicians to gain a broader understanding of clinical diagnoses, treatments, and outcomes of their cases.

## Conclusions

This report describes an MFS case with the unusual complication of scapular winging following thoracotomy for right thoracic scoliosis. Attention needs to be paid to surgical techniques in patients with hyperlaxity, especially in MFS.

## Data Availability

Not applicable.
